# Comparative Sealing Ability of Different Materials for Repair of Iatrogenic Perforations: An In Vitro Study

**DOI:** 10.1155/ijod/2650606

**Published:** 2026-09-30

**Authors:** Miranda Stavileci, Astrit Kuçi, Agime Dragidella

**Affiliations:** ^1^ Faculty of Medicine Department of Dental Pathology and Endodontics University of Prishtina Prishtina Kosovo uni-pr.edu; ^2^ Faculty of Dentistry Department of Dental Pathology and Endodontics AAB College Prishtina Kosovo aab-edu.net

**Keywords:** amalgam, biodentin, glass ionomer cement, MTA, root perforation

## Abstract

**Objective:**

The aim of this in vitro study was to assess and compare the sealing ability of root perforation repair materials: Biodentin, mineral trioxide aggregate (MTA), glass ionomer cement (GIC), and amalgam in extracted human teeth, using stereomicroscope.

**Material and Methods:**

For this in vitro study were tested 50 extracted mandibular molars with artificial perforations and were randomly divided into four experimental groups (*n* = 10) according to material used. Ten samples served as positive and negative control groups (*n* = 5, for each control group). Following perforation sealing, all samples were stored and after dye exposure were rinsed and dried. After their longitudinally sectioned through the center of repaired material, the extent of dye penetration at the perforation site was measured with stereomicroscope. Data were analyzed using one‐way ANOVA and Kruskal–Wallis tests, followed by Bonferroni post hoc comparisons, with significance set at *p* < 0.05.

**Results:**

The samples repaired with Biodentin demonstrated the lowest mean dye leakage, followed by MTA group, amalgam, and GIC group. Statistical analysis showed significant differences among tested groups (*p* < 0.05). Post‐hoc comparisons revealed that Biodentin group and MTA showed significantly superior sealing ability than GIC group, while no statistical differences between Biodentin and MTA group.

**Conclusions:**

The findings of this in vitro study under the tested conditions showed that Biodentin and MTA provided better sealing ability in root perforation repair. Amalgam showed intermediate performance, while GIC showed the highest level of micro leakage.

## 1. Introduction

Endodontic treatment can be associated with complications caused by anatomical variations, procedural difficulties, or biological factors [1]. Perforation, an artificial opening between the root canal system and the periodontal tissues, is among the most common complications that may occur during root canal treatment [2]. This complication can occur as a result of pathological processes, such as dental caries or root resorption, or as an operative procedural accident. Specifically, iatrogenic root perforations may occur during endodontic access cavity preparation, root canal preparation using instruments, or preparation for post‐application [3].

According to previous investigations, accidental root perforations occur in ~2%–12% of endodontically treated teeth [4, 5]. Infection can occur after perforation at either the perforation site or in the root canal or periodontal tissues, which may impair healing and initiate an inflammatory process, resulting in pain and suppuration [6]. The most important factor affecting prognosis is the location of the perforation in relation to the level of epithelial attachment and the crestal bone [7]. The perforation site should be closed as soon as possible to prevent apical migration of the epithelial attachment and subsequent periodontal defects [8].

Numerous factors may cause perforation of the pulp chamber, such as calcifications, plunging of a bur through the thin floor, or inadequate removal of the pulp chamber roof, leading to misdirection of the burr during access preparation [9].

Perforation defects can be repaired using nonsurgical endodontic or surgical techniques. Successful perforation repair depends on the ability to seal the perforation and reestablish a healthy periodontal ligament. Various materials have been used for perforation repair, such as amalgam, indium foil, gold foil, zinc oxide eugenol, Super EBA, IRM, Cavit, glass ionomer cement (GIC), composite, dentin chips, tricalcium phosphate cement, hydroxyapatite, calcium hydroxide, calcium‐enriched mixture, mineral trioxide aggregate (MTA), and Biodentin [10, 11]. Materials used for managing perforations should exhibit biocompatibility, good adherence to tooth structures, bactericidal properties, and non‐toxicity and non‐resorb ability. In addition, the material should be easy to manipulate and apply clinically. Most importantly, such materials must ensure an effective hermetic seal to prevent micro leakage at the perforation site [12, 13].

In recent years, bio ceramic materials used in endodontics have mainly been composed of aluminum, zirconium, and bioactive glass, all of which exhibit important biological properties. These materials are well tolerated by tissues because they are nontoxic, chemically stable, and do not shrink after setting [14]. Additionally, an important feature of bio ceramics is their ability to form a hydroxyapatite layer once they are set, which enhances their interaction with the surrounding dental tissues, supports the healing process, promotes cementoblast activity, demonstrates excellent biocompatibility with periapical tissues, and exhibits favorable sealing properties when used to manage root perforations [15, 16].

The determination of micro leakage is a critical parameter for research and clinical evaluations of sealing materials that are directly used in root perforation areas [17, 18].

Micro leakage assessment includes different leakage models: dye extraction (DE), dye penetration, bacterial leakage models, fluid filtration, and advanced imaging methods. According to Camps and Pashley [19], the DE and dye penetration assessment techniques demonstrate results similar to those of liquid filtration; however, they are simpler methods and less time‐consuming, which make them practical choice for assessing micro leakage in endodontic’s investigations. Although bioactive materials have shown favorable outcomes in root perforation treatment, the perforation location on treatment outcomes is still not fully understood. Comparative data on sealing ability of repair materials, their micro leakage resistance, and adaptation under in vitro conditions can contribute to a better understanding of their performance. Although several materials are available for root perforation repair, evidence directly comparing their sealing ability remains limited. MTA and Biodentin are commonly used calcium silicate‐based materials, while GIC and amalgam have traditionally been used for this purpose. As an effective seal is important for preventing leakage and supporting successful perforation repair, a direct comparison of these materials under standardized conditions may provide useful information for clinical material selection. Therefore, this study aimed to compare the sealing ability of MTA, Biodentin, GIC, and amalgam for root perforation repair in extracted human teeth using a stereomicroscope.

Null hypothesis (*H*_0_): There is no statistical significant difference in micro leakage among root perforation treated with Biodentin, MTA, GIC, and amalgam.
Alternative hypothesis (*H*_1_): There is a statistically significant difference in micro leakage among root perforation treated with Biodentin, MTA, GIC, and amalgam.

## 2. Material and Methods

This study was approved by the Ethics Commission of the University Dentistry Clinical Center of Kosova (Nr.734). In this study, we evaluated 50 human mandibular molars that were extracted for periodontal reasons. Extracted human mandibular molars with fully developed roots and no visible structural defects were selected for this study. Teeth with severe anatomical variations, open apices, extreme curvatures, no endodontic treatment, or crown and root fractures were excluded.

The sample size was calculated using the power analysis performed by 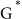 Power software (version 3.1.9.7). This calculation was based on one‐way ANOVA used test, assuming a large effect size (Cohen’s *f* = 0.40), the level of significance 0.05, and statistical power of 80%. So, to meet the calculated sample size requirement, a total of 50 extracted human teeth were included in the study.

The teeth were stored in a 0.1% thymol solution until the initiation of the experiment.

A standardized endodontic access cavity was prepared for all samples, followed by the identification of all canal orifices and the negotiation of the root canals to the apical length. The canal orifices were sealed with composite fillings, and a standardized artificial perforation was created in the center of the pulpal floor, at the furcation area of each molar, using a round bur under water cooling.

After sample preparation, all teeth were numbered consecutively and randomly assigned to one of four experimental groups (10 teeth per group) according to the tested repair material. This random allocation was performed before the experimental procedure to minimize selection bias and ensure the distribution of samples among the experimental groups.

All materials were prepared and strictly used in accordance with the manufacturer’s instructions. In addition, no repair material was placed in the positive control group, while in the negative control group, the entire tooth surface, including the perforation area, was coated with nail varnish to prevent dye penetration.

Perforation defects with a diameter of 1 mm and depth of 2 mm were created with sterile round bur same size (#12). The same‐sized 1 mm bur was used for all samples, while the depth was controlled at 2 mm using a premeasured reference mark on the bur. The same preparation procedure was followed for all samples to minimize variations related to the natural anatomy of the furcation region.

All perforations were prepared by the same operator to minimize procedural variability and after this were repaired using four different materials: Biodentin (Septodont, Saint Maur des Fosses Cedex, France), Gr.1; MTA (Dentsply Tulsa Dental, Tulsa, OK, USA), Gr.2; GIC (Securafil, Hamburg, Germany), Gr.3; and amalgam (Cavex Non Gamma‐2), Gr.4.

All materials were prepared in accordance with the manufacturer’s instructions and placed into the perforation using appropriate instruments. MTA and Biodentin were condensed with a hand plugger, GIC was adapted with a plastic filling instrument, and amalgam was condensed with an amalgam condenser. Each material was placed to completely fill the perforation defect, providing a ~2 mm thickness of repair material. No internal matrix was used, and excess material was removed with a small excavator to obtain a uniform surface at the level of the pulpal chamber floor.

To assess micro leakage, the external surface of each tooth was coated with two layers of nail varnish, leaving a standardized 1–2 mm area around the furcation region uncoated. Each layer was allowed to dry completely before the next layer was applied.

Following repair, all access cavities were restored with a composite resin (Gradia Direct) to simulate clinical conditions. The specimens were then stored in an incubator (Innovens, Jouan, France) at 37°C and 100% humidity for 24 h to allow complete setting of the repair materials (as an initial set occurs in several minutes to several hours). The dye leakage approach in this study was justified by its sensitivity and widespread acceptance in endodontic research [19].

Subsequently, all samples were immersed in a 2% methylene blue dye solution for 48 h at room temperature. After dye exposure, the teeth were thoroughly rinsed under running water to remove any residual dye, and the samples were then air‐dried. Each tooth was longitudinally sectioned at the same longitudinal plane, passing through the center of the repaired perforation using a diamond or a carborundum disc under copious water cooling to prevent heat generation and structural damage. The extent of dye penetration at the perforation site (Figures 1–4), an indicator of sealing ability, was evaluated using a stereomicroscope at 25× magnification (Brunel MX‐6& UK), at a single time point, which was considered sufficient to assess the initial sealing ability of tested materials [20]. The examiners who measured dye penetration were blinded to the group allocation in order to minimize evaluation bias. To assess the reliability of the measurements, inter‐ examiner agreement was calculated using Cohen’s kappa coefficient. The agreement between the two examiners was excellent (Cohen’s *k* = 0.87).

**Figure 1:**
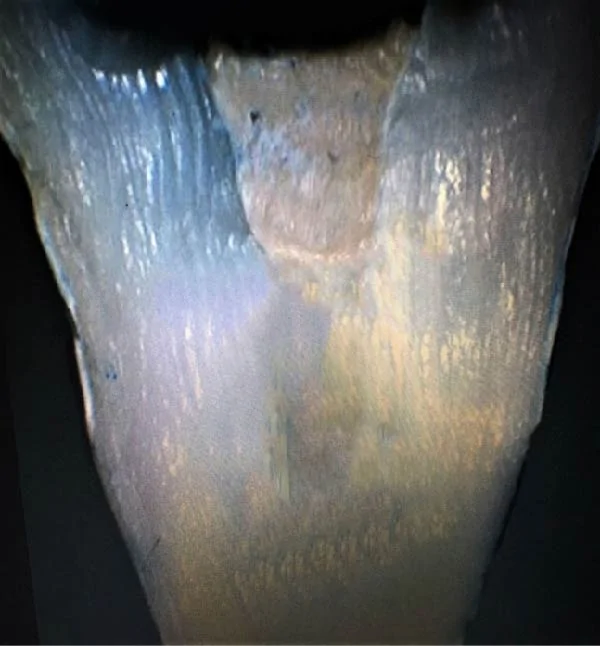
Dye penetration in sample treated with Biodentin.

**Figure 2:**
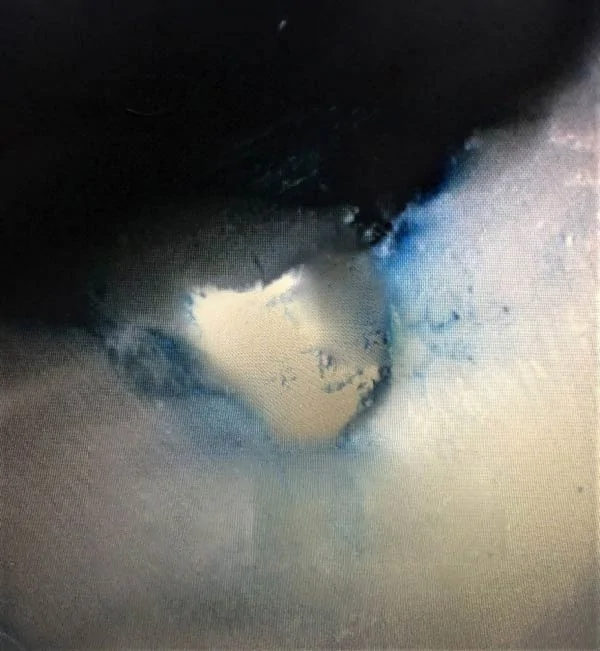
Dye penetration in perforation repaired with MTA.

**Figure 3:**
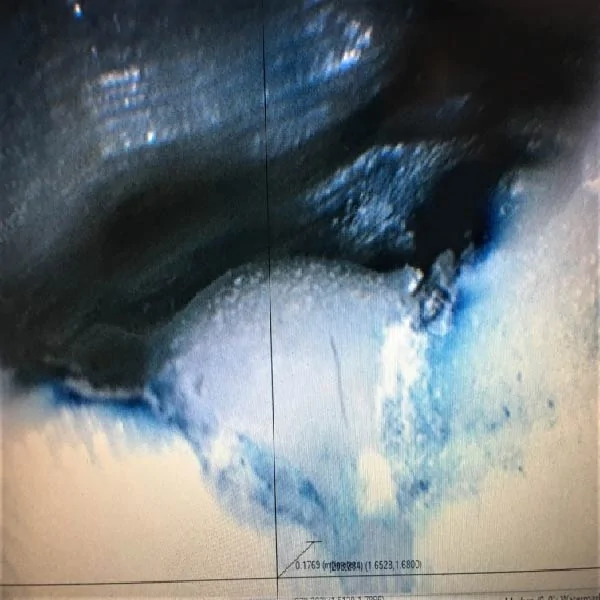
Dye penetration in perforation repaired with GIC.

**Figure 4:**
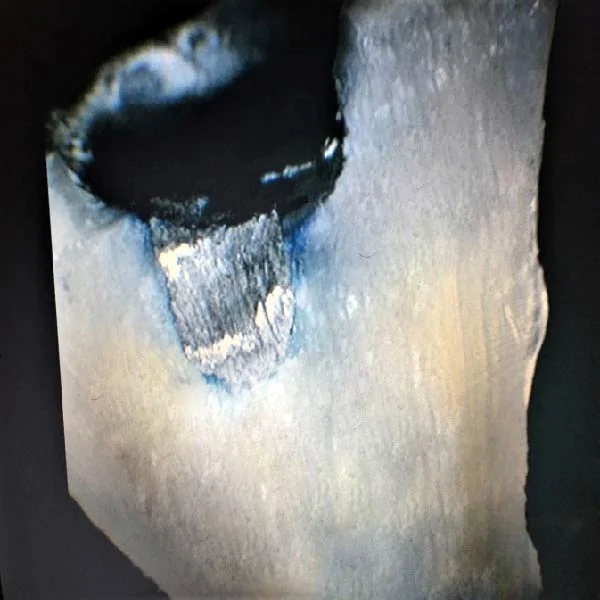
Dye penetration in perforation repaired with amalgam.

The extent of dye penetration at the perforation site was measured linearly from the external margin of perforation toward the internal place of the repair material. The extent of dye penetration at the perforation site was measured in millimeters.

Data were collected and analyzed statistically using analysis of variance (ANOVA), Kruskal–Wallis, and Bonferroni tests. The level of significance was set at *p* < 0.05 and *p* < 0.001.

## 3. Results

The extent of dye penetration at the perforation site (mm) was used as a quantitative measure of micro leakage across the four experimental groups: Biodentin, MTA, GIC, and amalgam (*n* = 10 per group). Descriptive statistics demonstrated that the lowest mean dye penetration at the perforation site was observed in the Biodentin group (0.15 mm), followed by the MTA group (0.24 mm). Higher values were recorded for the amalgam (0.92 mm) and GIC groups (1.18 mm), indicating greater micro leakage (Table 1 and Figure 5).

**Figure 5:**
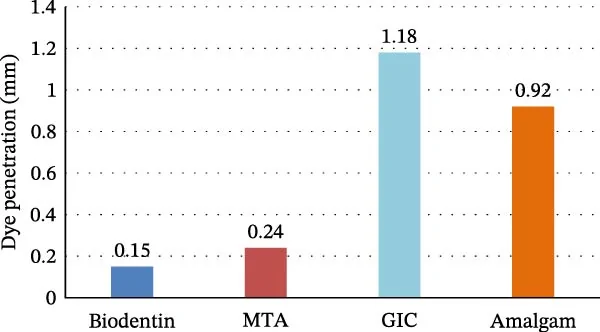
Dye penetration at perforation site (mm) according to experimental groups: Biodentin showed the lowest mean micro leakage (0.15 mm), followed by MTA (0.24 mm), amalgam (0.92 mm), and GIC (1.18 mm).

**Table 1:** Dye penetration (mm) according to four perforation repair materials.

Experimental group	Mean ± SD (mm)	Median (IQR) (mm)	Bonferroni pairwise comparisons
Biodentin (*n* = 10)	0.15 ± 0.14	0.11 (0.05–0.20)	vs. MTA, *p* > 0.05; vs. GIC, *p* = 0.008; vs. amalgam, *p* = 0.032
MTA (*n* = 10)	0.24 ± 0.18	0.20 (0.10–0.32)	vs. GIC, *p* = 0.008; vs. amalgam, *p* = 0.041
Glass ionomer cement (GIC) (*n* = 10)	1.18 ± 0.30	1.20 (1.00–1.35)	vs. amalgam, *p* = 0.032
Amalgam (*n* = 10)	0.92 ± 0.25	0.90 (0.75–1.10)	—

*Note:* Statistical analysis: one‐way ANOVA followed by Bonferroni post hoc test.

Abbreviations: GIC, glass ionomer cement; MTA, mineral trioxide aggregate; SD, standard deviation.

Prior to inferential testing, the data distribution was assessed. The normality of the data distribution was assessed using the Shapiro–Wilk test. Intergroup comparisons of average dye penetration were performed using ANOVA.

Given the small sample size and non‐normal distribution tendencies, both parametric (one‐way ANOVA) and non‐parametric (Kruskal–Wallis) tests were applied to ensure robustness of the findings. One‐way ANOVA revealed a statistically significant difference in the mean dye penetration among the four groups (*p* < 0.001), indicating that at least one material differed significantly in sealing ability. This finding was further confirmed by the Kruskal–Wallis test, which also demonstrated a statistically significant difference between the groups (*p* < 0.001).

To identify pairwise differences, post hoc analysis with Bonferroni correction was performed. Biodentin vs. MTA showed no statistically significant difference (*p* > 0.05), suggesting a comparable sealing performance. Biodentin vs. GIC showed a statistically significant difference (*p* = 0.008), with Biodentin showing a superior sealing ability. Biodentin vs. amalgam showed a statistically significant difference (*p* = 0.032) favoring Biodentin. MTA vs. GIC showed a statistically significant difference (*p* = 0.008), with MTA demonstrating lower micro leakage. MTA vs. amalgam showed a statistically significant difference (*p* = 0.041) favoring MTA. GIC vs. amalgam showed a statistically significant difference (*p* = 0.032), with amalgam showing slightly better sealing than GIC.

As expected, the positive control group demonstrated complete dye leakage, whereas no dye penetration was reported in the negative control group, confirming the viability of the experiment.

## 4. Discussion

Preventing micro leakage is a key goal in the management of root perforations because the penetration of fluids and microorganisms can compromise treatment outcomes [21]. Accordingly, materials capable of blocking small dye molecules are reported to be effective in preventing the penetration of larger particles such as bacteria [22]. Therefore, evaluating dye leakage remains a widely used and reliable method for assessing the sealing ability of endodontic materials [23]. In particular, the dye penetration technique allows quantitative assessment by measuring the amount of dye that penetrates the material interface. Because dye molecules are smaller than bacteria, their ability to infiltrate even minimal gaps makes this method highly sensitive and clinically relevant [24, 25].

While previous investigations have demonstrated promising results as root perforation repair materials [12], their sealing ability evidence still remains limited. So, to address this gap, we tested four repair materials under standardized in vitro conditions.

In this study, 2% methylene blue was used as the tracer dye because of its small molecular size and high diffusion capacity. Therefore, previous authors, such as Brandao et al. [26], have emphasized that methylene blue provides a more precise evaluation of micro leakage than bacterial models due to its low molecular weight.

Regardless of their cause or location, root perforations can negatively influence the success of endodontic treatment. However, when the perforation is adequately sealed, the prognosis improves significantly [27]. In our study, the extent of dye penetration at the perforation site was measured in millimeters under a stereomicroscope to evaluate leakage between the repair material and the tooth structure. Biodentin showed the lowest mean dye leakage (0.15 mm), followed by MTA (0.24 mm), whereas amalgam (0.92 mm) and GIC (1.18 mm) demonstrated considerably higher leakage values. The findings of the present study are consistent with those of several previous reports. Abimanyu et al. [28] found no significant difference between Biodentin and MTA in terms of dye leakage, suggesting that both materials provide comparable sealing properties. Similarly, Panwar and Hada [29] reported no statistically significant difference among Biodentin, MTA, and zirconia‐reinforced GIC for furcation perforation repair.

However, other studies have suggested superior performance of Biodentin. Hegde et al. [30] reported better sealing abilities with Biodentin than with MTA, whereas Desai et al. [31] demonstrated significantly lower micro leakage values for Biodentin (0.38 ± 0.27 mm) than for MTA (1.1 ± 0.42 mm). In a study conducted by Mohan et al. [32], the lowest level of micro leakage was observed in the Biodentin group (0.88 ± 0.02), followed by the ProRoot MTA group (1.24 mm ± 0.13), while the highest dye penetration was recorded in the resin‐modified GIC group (3.62 mm ± 0.46).

Nevertheless, MTA remains a well‐established and effective material. Nazeer et al. [33] highlighted its robust performance as a reparative material for iatrogenic perforations, confirming its long‐standing role as a reliable treatment option. In contrast, the higher leakage observed with amalgam and GIC in the present study aligns with their known limitations, particularly their higher solubility and lower effective sealing capacity.

Overall, the results of this study have rejected the null hypothesis, whereas they have accepted the alternative hypothesis. The current results support the use of bio ceramic‐based materials for perforation repair, with Biodentin showing a tendency toward better sealing performance.

One possible mechanism for better sealing performance observed with Biodentin under the conditions of this in vitro study could be potentially explained to its favorable physicochemical properties, including its flow, solubility, and structural characteristics [34, 35], although these factors were not directly evaluated in the present study. In contrast, although MTA is widely used and highly biocompatible, it’s setting reaction produces larger amounts of calcium hydroxide, which may result in a less compact structure and slightly increased solubility [35].

Both MTA and Biodentin have the potential to effectively restore the biomechanical properties of perforated roots [36]. Root perforation repair and backfilling with these bioactive cements can reinforce the tooth [37].

As an in vitro study, the experimental conditions could not fully imitate the complex biological environment found in vivo, including the presence of blood, tissue fluids, inflammation, and functional loading. Other limitations include the use of the dye penetration method, the short observation period, and possible anatomical differences among the extracted teeth. In addition, only one perforation size and location were assessed at a single time point.

Nevertheless, given the variability in findings across different studies, further well‐designed studies are needed to confirm these results and guide clinical decision‐making.

## 5. Conclusions

With the limitations of this in vitro study, it may be concluded:

• Biodentin and MTA reported the lowest levels of dye penetration at the perforation site among the tested materials, suggesting better sealing ability under the conditions of this in vitro study.
• Although Biodentin exhibited a slightly lower mean of dye penetration at the perforation site than MTA, the difference between the two materials was not statistically significant.
• GIC and amalgam showed significantly greater dye penetration at the perforation site than Biodentin and MTA in this experimental model.

## Author Contributions

Miranda Stavileci contributed in conceptualization, methodology, validation, investigation, data curation, writing – original draft. Astrit Kuçi contributed in data interpretation. Agime Dragidella contributed in writing – original draft, review and editing.

## Funding

This research received no external funding.

## Disclosure

All authors have read and approved the final version of the manuscript.

## Conflicts of Interest

The authors declare no conflicts of interest.

## Data Availability

The datasets generated and analyzed during the current study are obtainable from the corresponding author upon reasonable request.
